# Before the bombing: High burden of traumatic injuries in Kunduz Trauma Center, Kunduz, Afghanistan

**DOI:** 10.1371/journal.pone.0165270

**Published:** 2017-03-10

**Authors:** Hamayoun Hemat, Safieh Shah, Petros Isaakidis, Mrinalini Das, Nang Thu Thu Kyaw, Sattar Zaheer, Abdul Qayeum Qasemy, Mutallib Zakir, Gbane Mahama, Catherine Van Overloop, Lynette Dominguez

**Affiliations:** 1 Médecins Sans Frontières–Operational Center Brussels, Kabul, Afghanistan; 2 Médecins Sans Frontières–Operational Research Unit, Luxembourg City, Luxembourg; 3 International Union against Tuberculosis and Lung Disease, Mandalay, Myanmar; 4 Médecins Sans Frontières–Operational Center Brussels, Kunduz, Afghanistan; 5 Médecins Sans Frontières–Operational Center Brussels, Brussels, Belgium; Azienda Ospedaliero Universitaria Careggi, ITALY

## Abstract

**Background:**

Médecins Sans Frontières (MSF) has been providing healthcare in Afghanistan since 1981 including specialized health services for trauma patients in Kunduz Trauma Center (KTC) from 2011. On October 3^rd^, 2015, a US airstrike hit the KTC, killing 42 people including 14 MSF staff. This study aims to demonstrate the impact on healthcare provision, after hospital destruction, by assessing the extent of care provided for trauma and injuries by the MSF KTC and to report on treatment outcomes from January 2014 to June 2015, three months prior to the bombing.

**Methods:**

This is a descriptive, retrospective review of hospital records. All patients with traumatic injuries registered in the Emergency Department (ED) or hospitalized in In-Patients Department (IPD) and/or Intensive Care Unit (ICU) of KTC between January 2014 and June 2015 were included in the study.

**Results:**

A total of 35647 patients were registered in KTC during the study period. 3199 patients registered in the ED were children aged <5 years and 310 of them were admitted including 47 to the ICU. 77.5% patients were from Kunduz province and the remaining were from other provinces. The average length of stay was 7.3 days and 3.3 days while the bed occupancy rate was an average 91.1% and 75.8% in IPD and ICU, respectively. Of 4605 IPD patients, 105 (2.3%) developed complications. Among those admitted to the ICU, 12.6% patients died. About one-third surgical interventions were carried out on an urgent basis and the major proportion (45.8%) of surgical procedures was wound surgery followed by orthopedic surgery (27.0%).

**Conclusions:**

This study highlights the high burden of traumatic injuries in Kunduz province and MSF Trauma Center’s contribution to saving lives, preventing disabilities and alleviating suffering among adults and children within the region. The bombing and destruction of KTC has resulted in a specific gap in critical healthcare services for the local communities in the health system of this war-ravaged region. This suggests the urgent need for reconstruction and re-opening of the center.

## Introduction

Afghanistan is a landlocked country, ravaged by 3.5 decades of conflict which has debilitated the health infrastructure[1]. Some regions have been more affected by civil war than others, especially the southern region, while other regions have been affected by natural disasters including earthquakes, landslides, avalanches and floods. Natural and man-made disasters have crippled the government from catering to all types of trauma, including injuries associated with violence (e.g. assault, torture, rape, gun shots, landmines, bombs), and unintentional injuries not related to violence (e.g. road traffic accident, building collapses, falls etc).

Despite substantial reliance on external humanitarian assistance, ongoing insecurity has limited the ability of international organizations to provide medical care[2]. As a consequence there are limited data available and very few reports of humanitarian assistance programs in the country. A large proportion of the population does not have access to health services, also due to security problems and unavailability of public transportation[3][4]. Emergency health services, including trauma care, are limited and difficult to reach[5].

Médecins Sans Frontières (MSF) has been providing healthcare in Afghanistan since 1981[6] (with a 5-years gap between 2004 and 2009 after national and international staffs were killed[7]). Since 2009 MSF has been providing health services in Kunduz, Khost, Helmand, and Kabul provinces[6][8]. In 2011, an 80-beds trauma center was opened in Kunduz, in the north of the country providing free health services to trauma patients [9]. On October 3^rd^, 2015, a US airstrike hit the trauma center in Afghanistan; 42 people, including 14 MSF staff, were killed [10]. The attack was a violation of International Humanitarian Law and the Geneva Conventions [11,12]. Since then the Kunduz Trauma Center remains closed and the needs of the population are unmet.

The aim of this study was to demonstrate the impact on provision of healthcare after hospital destruction by assessing the extent of care provided for trauma and injuries by the MSF Kunduz Trauma Center (KTC) and to report on patient characteristics and outcomes from January 2014 to June 2015, three months before the hospital was bombed and destroyed.

## Methods

### Study design

This study is a retrospective, descriptive study, which utilizes patient data collected during routine operations of the different departments within the hospital.

### Setting

#### General setting

Kunduz province is located in the northern half of Afghanistan with an approximate population of 1,204,400, administratively divided into seven districts. The province has a tribal lifestyle, which is historically how people in this region live. The government has no control on this entire province and even in urban centers, the level of insecurity is a major concern for civilians; kidnappings, armed robberies, random killings, targeted bomb blasts, roadside bomb blasts and suicide attacks, being rife. Due to security concerns and lack of accountability, humanitarian organizations are wary of providing aid in any form. Therefore, in the past, as well as currently, there is an extreme gap of healthcare service availability[4].

#### Kunduz Trauma Center (KTC)

From 2011, MSF was providing specialized health services free of charge in the 80-bed KTC: the capacity was increased to 92 beds in 2015, since the services were well accepted by the locals, before the hospital was bombed in 2015.

Sixty percent of the patients were from Kunduz district, eighteenth percent of the patients were coming from peripheral districts of Kunduz province while twenty two percent of them were from other provinces.

KTC had an Emergency Department (ED), which utilized the South African Triage Scale (SATS). The KTC included three Operating Rooms (ORs). The In-Patient Department (IPD) had 92 beds and included an 8-beds Intensive Care Unit (ICU).

Physiotherapy services were offered by trained staff to admitted patients as well as outpatients (discharged from the hospital). Mental health services including counseling were offered to admitted and discharged patients, especially to those suffering from intentional injuries and to victims of violence. A fully functional on-site microbiology laboratory was providing culture and Drug-Sensitivity Testing (DST) for Surgical Site Infections (SSI). Patients in need of prostheses were referred to specialized centers in Kunduz or to Kabul.

An Out-Patient Department (OPD) was available–by appointment–only to patients who required follow-up, dressing, debridement, counseling and physiotherapy, post-discharge. Patients who missed follow-ups were contacted via mobiles by the OPD staff.

A health promotion team conducted health promotion sessions at IPD, ED, and OPD for the patients and care takers.

### Study population

All patients with traumatic injuries registered in the ED or admitted in IPD and/or ICU Kunduz Trauma Center between Jan 2014 to June 2015 have been included in the study.

### Data collection and analysis

The KTC used standardized, paper-based registers as well as electronic databases. Data quality checks were regularly performed to minimize errors in data entry and ensure data validity and consistency. For this study relevant individual patient level data from both sources were extracted and entered into an Excel database. Variables include age, gender, registration/admission/discharge dates, type of trauma, operation type and urgency, and procedure(s) performed. Trauma included injuries associated with violence (e.g. assault, torture, rape, gun shots, landmines, bombs), and unintentional, non-violence-related injuries (e.g. road traffic accident, building collapses, falls etc.). Procedures are grouped into the following categories: minor surgery, wound surgery, specialized surgery, visceral surgery, and orthopaedic surgery. Outcomes include treatment and discharge, referral, death, LAMA (left against medical advice) and for ED patients admission to IPD/ICU. Descriptive statistics (numbers and proportions) are used in the analysis.

### Ethics

Permission for the study was obtained from the Institutional Review Board (IRB) of Ministry of Public Health, Kabul, Afghanistan and the MSF Ethics Review Board (ERB), Geneva, Switzerland. As this was a study of routinely collected monitoring data, informed consent from the patients was not obtained. The named ethics committees specifically approved the study and waived the need for consent.

## Results

A total of 35647 patients were registered in KTC from January 2014 to June 2015; 77% of them were male. The main demographic characteristics of patients registered in the different departments of KTC are summarized in Table 1.

**Table 1:** Demographic characteristics of patients with traumatic injuries registered in KTC, Kunduz, Afghanistan from January 2014—June 2015.

Characteristic	Patients, n (%)			
	ED	OD	IPD	ICU
**Total**	**35647**	**3722**	**4605**	**617**
**Age (in years)**				
<5	3199 (8.9)	131 (3.5)	310 (6.7)	47 (7.6)
5–19	1653 (41.0)	1382 (37.1)	1833 (40.0)	240 (38.8)
20–50	15197 (42.6)	1915 (51.4)	2054 (44.6)	288 (46.7)
>50	2657 (7.5)	294 (7.9)	408 (8.9)	42 (6.8)
**Sex**				
Male	27277 (76.5)	514 (86.2)	3837 (83.3)	100 (83.5)
Female	8370 (23.5)	3208 (13.8)	768 (16.7)	517 (16.5)
**Origin**				
Kunduz province	27654 (77.6)	_____	_____	_____
Other provinces	7993 (22.4)	_____	_____	_____

ED = Emergency Department; OD = Operation Department; IPD = In-Patient Department; ICU = Intensive Care Unit.

**Fig 1:**
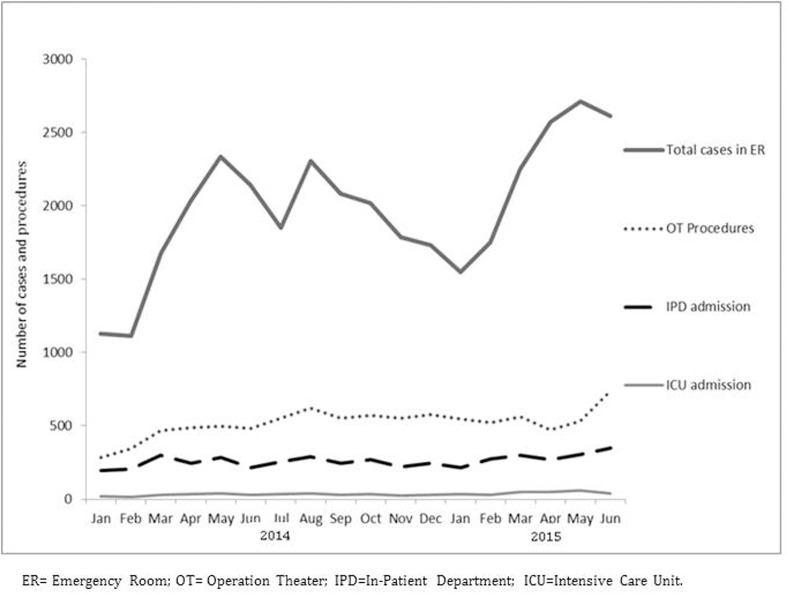
Number of cases registered and number of procedures done in different departments in KTC, Kunduz, Afghanistan from January 2014—June 2015.

A significant proportion of patients were children and adolescents ranging from 41% of the operated patients to 50% of the patients registered in the ED (Table 1). There were 3199 children aged younger than 5 years registered in ED and 310 of them were admitted to KTC IPD including 47 to the ICU. During the study period, 27654 (78%) patients were from the Kunduz province, while the remaining were from other provinces. Fig 1 shows the trend of traumatic injury cases registered in each department. A higher number of patients showed up at the ED during the second quarter, of both 2014 and 2015 and the overall number of ED consultations showed an increase, over time.

**Fig 2:**
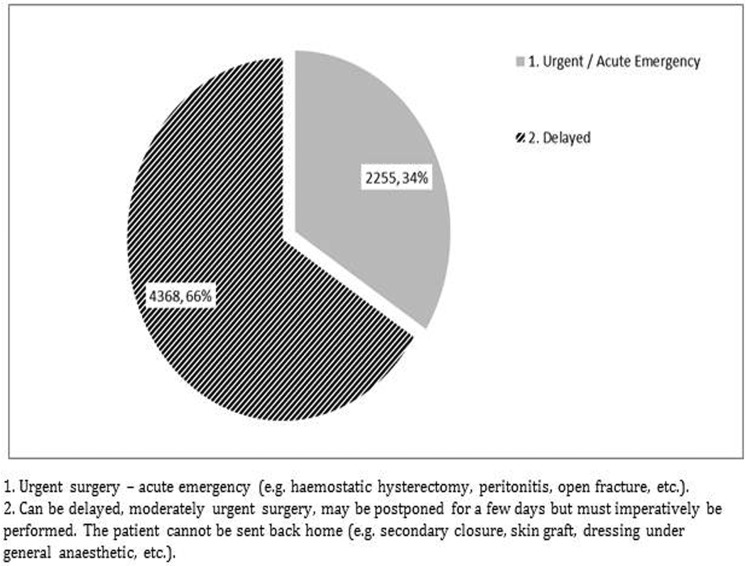
Degree of urgency for different types of surgical interventions in Operation Department, KTC, Kunduz, Afghanistan from January 2014—June 2015

In terms of order and urgency, about 34% (2255) of surgical interventions were carried out immediately (Fig 2). Table 2 summarizes the total number and type of operations performed. The major proportion (46%) of surgical procedures included wound surgery, with orthopedic surgery accounting for 27%.

**Table 2:** Number and type of surgical interventions in Operation Department, KTC, Kunduz, Afghanistan from January 2014—June 2015

**Surgical Interventions (N = 9369)**	**n**	**(%)**
**Minor surgery**	1822	19.4
Tube thoracostomy	137	7.5
**Wound surgery**	4294	45.8
Extensive Debridement, fasciotomy	3925	91.4
**Specialized surgery**	163	1.7
Neurosurgery	79	48.5
Thoracotomy	15	9.2
Vascular surgery	47	28.8
**Visceral Surgery**	550	5.9
Exploratory Laparotomy	347	63.1
**Gynecology & Obstetrics**	6	0.1
**Orthopedic surgery**	2534	27
Amputation of limbs	190	7.5
Fracture reduction	580	22.9
External Fixation	711	28.1
Other	174	6.9
**Internal fixation ^$^**	879	34.7
Plates	53	25.6
Intramedullary nailing	60	29
Dynamic Hip Screw	8	3.9
Others	86	41.5

^**$**^ data for sub type of Internal fixation is only from first six months of 2015.

In IPD the Average Length of Stay (ALoS) was 7.3 days while the Bed Occupancy Rate (BOR) was, at an average: 91%. ALoS in ICU was 3.3 days and an average of 76% of the ICU beds was occupied during the study period. Of 4605 IPD patients, 105 (2%) developed complications. Mortality among the patients admitted to ICU was 13%. The outcomes recorded in each department are summarized in Table 3.

**Table 3:** Outcomes of patients with traumatic injuries registered in different departments in KTC, Kunduz, Afghanistan from January 2014-June 2015

Outcomes	ED n (%)	IPD n (%)	ICU n (%)
**Treated & Discharged**	28522 (78.9)	4362 (94.7)	516 (82.3)
**Referred**	2631 (7.3)	83 (1.8)	11 (1.8)
**Left against medical advice**	399 (1.1)	113 (2.4)	21 (3.3)
**Dead**	30 (0.1)	47 (1.0)	79 (12.6)
**Hospitalized**	4605 (12.8)	-	-
**Total**	**36187**	**4605**	**627**

## Discussion

This is the one of the first studies of the Kunduz Trauma Center, a large specialized facility in northern Afghanistan that provided care to a large number of injured adults and children in the region.

More than eight percent of patients registered in the KTC were children under five. This is only representative of the patients who sought care at KTC, and no population level inferences can be made from this study, which is a limitation of this study design. The main age groups of patients registered in the different departments of KTC were young adults and adults between 20 to 50 years. This age group is particularly affected by synonymous violent injuries globally as it is more likely to be involved in fighting, and being mobile, is also at higher risk of non-violent traumatic injuries[13].

The predominance of male gender among the KTC patients most likely reflects the context; women in Afghanistan are often at home, their movements are limited and therefore they may be at a lower risk for intentional and unintentional injuries than males. Nevertheless, women and children are extremely vulnerable during escalation of violence and assault, rape and traumatic injuries can become common, and remain undocumented [14].

Although access to basic healthcare might be limited for women and girls in Afghanistan; however, access to trauma care has been anecdotally reported to be less challenging. The extent of care provided in KTC was steadily increasing from 2014 to 2015, with a rather clear seasonal variation. ED visits increased from March to June as the weather conditions facilitated access to health services. In the same period escalation of violence was also recorded.

The great majority of patients treated in the ED were successfully treated and discharged to return home. Almost ten percent of patients from ED were referred to other health facilities, either due to a lack of space in KTC or due to specialized healthcare requirements. The mortality ratio within the ICU was relatively high at 13%, comparable to 10% reported from a Médecins Sans Frontières ICU in Tabarre Trauma Center in post-earthquake Haiti[15]. According to a study of 42 ICUs carried out several years ago, the unadjusted in-hospital mortality rates varied from 6.4% to 40%; and 90% of this variation was attributable to patient characteristics at admission [16,17].

The number of orthopedic interventions at KTC were increasing over time. This increase was mostly recorded for amputations and internal fixations that required additional resources and specialized skills. A prospective, cohort study on internal fixation outcomes was planned for 2015–2016, but the unexpected hospital closure prevented the team from providing services better suited to the patients.

This study had certain strengths. We have studied all patients registered in a large trauma center in a conflict setting over a long period of time. We described in detail the burden of disease, the variety of specialized services needed, as well as patient outcomes. The study adhered to the STROBE guidelines for reporting of observational data[18].

However, our study had several limitations as well. Firstly, the data presented were taken from paper and electronic registers used for routine monitoring and evaluation rather than for research, therefore missing values and errors in coding might have been common. However, data entry was done by trained staff using standardized forms and definitions, and crosschecking and validation were part of the routine data management. Secondly, we did not fully describe procedures performed and services offered outside of the main hospital departments such as OPD, physiotherapy, mental health services, health promotion and laboratory, which were all important components of the comprehensive care provided by MSF. Thirdly facility-based data does not include the patients who were injured in the community, those with trauma who were not able to reach care, or who did not seek care from MSF KTC. Therefore, our data does not represent the total burden of trauma in the population. Fourth, our study data likely reflects the expected age and gender distribution of patients for the specific context but we are unable to infer the epidemiology of trauma in the population from hospital-based data; for which population-based surveys would be a more appropriate method.

Despite these limitations, our study highlights a proportion of the high burden of traumatic injuries in Northern Afghanistan, while describing the extent of services offered at the KTC, highlighting its contribution to saving lives, preventing disabilities and alleviating suffering among adults and children within the region. The recent bombing and closure of KTC resulted in a huge loss for the local communities’ leaving a gap in critical services for the health system of this war ravaged region; many preventable deaths and disabilities will occur in years to come, especially if the center does not become operational again soon. The re-opening of the center is therefore urgently required, however the strict enforcement of the International Humanitarian Law and the Geneva Conventions that regulates armed conflict and provide protection for civilians, the wounded and medical personnel is an essential precondition.
